# Phenethyl Isothiocyanate Suppresses Stemness in the Chemo- and Radio-Resistant Triple-Negative Breast Cancer Cell Line MDA-MB-231/IR Via Downregulation of Metadherin

**DOI:** 10.3390/cancers12020268

**Published:** 2020-01-22

**Authors:** Yen Thi-Kim Nguyen, Jeong Yong Moon, Meran Keshawa Ediriweera, Somi Kim Cho

**Affiliations:** 1 Interdisciplinary Graduate Program in Advanced Convergence Technology and Science, Jeju National University, Jeju 63243, Korea; am20189301@jejunu.ac.kr; 2 Subtropical/Tropical Organism Gene Bank, Jeju National University, Jeju 63243, Korea; owenmjy@jejunu.ac.kr (J.Y.M.); meran@jejunu.ac.kr (M.K.E.); 3 Faculty of Biotechnology, College of Applied Life Sciences, SARI, Jeju National University, Jeju 63243, Korea

**Keywords:** phenethyl isothiocyanate, metadherin, cancer stem cells, resistance, reactive oxygen species

## Abstract

Resistance to chemotherapy and radiation therapy is considered a major therapeutic barrier in breast cancer. Cancer stem cells (CSCs) play a prominent role in chemo and radiotherapy resistance. The established chemo and radio-resistant triple-negative breast cancer (TNBC) cell line MDA-MB-231/IR displays greater CSC characteristics than the parental MDA-MB-231 cells. Escalating evidence demonstrates that metadherin (MTDH) is associated with a number of cancer signaling pathways as well as breast cancer therapy resistance, making it an attractive therapeutic target. Kaplan–Meier plot analysis revealed a correlation between higher levels of MTDH and shorter lifetimes in breast cancer and TNBC patients. Moreover, there was a positive correlation between the MTDH and CD44 expression levels in The Cancer Genome Atlas breast cancer database. We demonstrate that MTDH plays a pivotal role in the regulation of stemness in MDA-MB-231/IR cells. Knockdown of MTDH in MDA-MB-231/IR cells resulted in a reduction in the CSC population, aldehyde dehydrogenase activity, and major CSC markers, including β-catenin, CD44^+^, and Slug. In addition, MTDH knockdown increased reactive oxygen species (ROS) levels in MDA-MB-231/IR cells. We found that phenethyl isothiocyanate (PEITC), a well-known pro-oxidant phytochemical, suppressed stemness in MDA-MB-231/IR cells through ROS modulation via the downregulation of MTDH. Co-treatment of PEITC and N-Acetylcysteine (a ROS scavenger) caused alterations in PEITC induced cell death and CSC markers. Moreover, PEITC regulated MTDH expression at the post-transcriptional level, which was confirmed using cycloheximide, a protein synthesis inhibitor.

## 1. Introduction

Breast cancer remains the most frequently diagnosed cancer among women. Among the breast cancer sub-types, triple negative breast cancer (TNBC) is the most aggressive, accounting for 12–18% of all breast cancer cases. As TNBC cells lack the expression of hormone receptors (estrogen receptor and progesterone receptor) and human epidermal growth factor receptor 2 (HER-2), TNBC tumors do not respond to hormonal therapy [1]. Therefore, chemotherapy, radiotherapy, and surgery are widely used for the treatment of TNBC [2]. However, resistance to chemo and radiotherapies has been identified as a major obstacle in TNBC treatment.

Cancer stem cells (CSCs) possess an inherited capacity to self-renew and differentiate into distinct cancer cell types and display enhanced resistance to chemo and radiotherapies, making them extremely tumorigenic. Over the past decade, breast CSCs (BCSCs) have been identified as a promising target in breast cancer therapy [2,3]. The CSC markers CD44^+^/CD24^−^ are well-established surface bio-markers in BCSCs. CSCs are regulated through a range of key signaling pathways, such as Wnt/β-catenin, Notch, Hedgehog, phosphatase and tensin homolog (PTEN), and NF-κB, and irregularities in these signaling pathways are frequently found in CSCs [4]. Elevated aldehyde dehydrogenase (ALDH) levels and overexpression of ATP-binding cassette (ABC) transporters such as multidrug resistance protein 1 (MRP1)/ABCC1, multidrug resistance 1 (MDR1), P-glycoprotein (Pgp)/ABCC1, and breast cancer resistance protein (BCRP)/ABCG2/ABCP are commonly observed in CSCs [4]. Moreover, enhanced epithelial-to-mesenchymal transition (EMT) activity is recognized as a CSC marker [4]. Low reactive oxygen species (ROS) levels and elevated free radical scavengers have also been identified in CSCs [5], and ROS has been identified as an important target in the treatment of CSCs [6,7,8].

Among the various phytochemicals with anti-cancer effects, phenethyl isothiocyanate (PEITC) is one of the most prominent phytochemicals reported to control ROS levels in cancer cells [9]. Several studies have demonstrated that PEITC can exert pro-oxidant effects by depleting glutathione levels [10,11,12]. Moreover, a number of investigations have illustrated that PEITC can effectively target cancer cells by promoting the inhibition of cell proliferation and invasiveness [13,14,15], inducing apoptosis [16,17,18] and cell cycle arrest [19,20,21,22] in vitro and in vivo. However, studies on the potential anti-cancer efficacy of PEITC and its mechanism in CSCs, especially BCSCs, are extremely limited.

Metadherin (MTDH), also known as Lysine-rich CEACAM-1-associated protein (LYRIC) [23] or astrocyte elevated gene-1 (AEG) [24,25], is a tumor antigen mainly found in the cell membrane, cytoplasm, and nucleus [25]. Overexpression of MTDH has been reported in many cancer types, including liver [26,27], prostate [28,29], colorectal [30], glioblastoma [31], and breast cancers [32,33]. MTDH has been reported to regulate two distinct signaling pathways implicated in tumorigenesis and metastasis: The NF-κB [34,35] and mitogen - activated protein kinase (MAPK) [36,37] pathways. Several studies have indicated that MTDH can play a crucial role in the maintenance of low ROS levels [38] and CSC populations by regulating transcriptional factors such as Twist or β-catenin [31]. In addition, elevated expression of MTDH has been associated with breast cancer therapy resistance [33,39]. However, information on pre-clinical investigations assessing the effects of natural compounds on MTDH expression is extremely limited. The lack of pre-clinical data on MTDH targeting by natural products and the ability of PEITC to regulate ROS levels in CSCs led us to investigate the inhibitory effects of PEITC on stemness in our established chemo and radioresistant TNBC cell line, MDA-MB-231/IR.

## 2. Results

### 2.1. Stem Cell Characteristics of MDA-MB-231/IR Cells

**Figure 1:**
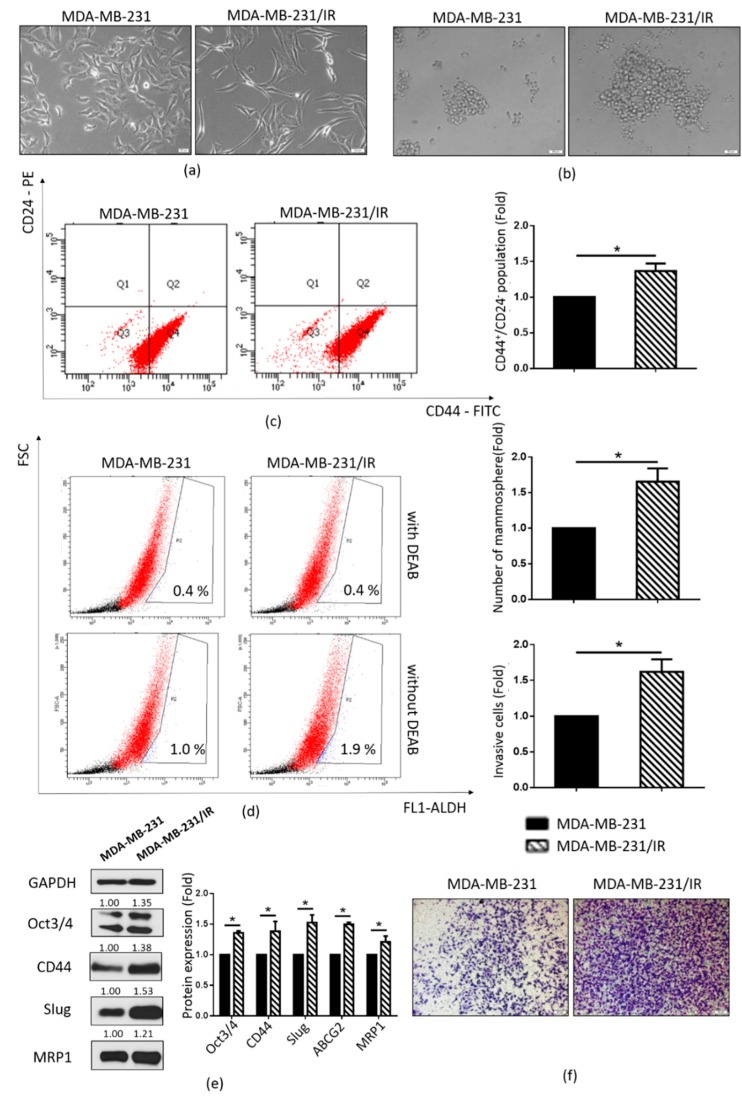
Characteristics of radioresistant MDA-MB-231/IR cells. (**a**) Morphologies of MDA-MB-231 and MDA-MB-231/IR cells cultured as monolayers (100× magnification). (**b**) MDA-MB-231 and MDA-MB-231/IR cells cultured as mammospheres in complete MammoCult Human Medium for 10 days (100× magnification). (**c**) CD44^+^/CD24^−^ population in MDA-MB-231/IR and MDA-MB-231 cells as analyzed by fluorescence-activated cell sorting (FACS). (**d**) ALDH^+^ population assessed by the ALDEFLUOR assay kit, with DEAB used as the negative control. (**e**) Cancer stem cell (CSC) markers were analyzed by Western blotting; * *p* < 0.05; results are presented as the mean ± standard deviation. (**f**) Invasion of MDA-MB-231/IR and MDA-MB-231 cells assessed by the Transwell cell invasion assay (100× magnification).

MDA-MB-231/IR cells were established in our laboratory by irradiating MDA-MB-231 cells at a dose of 2 Gy for 25 cycles [40]. In contrast to the parental cell line MDA-MB-231, MDA-MB-231/IR cells displayed mesenchymal-like morphology with a spindle-shaped appearance (Figure 1a). They also exhibited an enriched CSC population, as evidenced by the increased mammosphere formation capacity (Figure 1b), CD44^+^/CD24^−^ population (Figure 1c), and ALDH activity (Figure 1d). This observation was strongly supported by Western blot analysis, which showed increased expression of CSC markers such as CD44, Oct3/4, and MRP1 (Figure 1e). Moreover, there was a significant increase in the cell invasion capability of MDA-MB-231/IR cells compared to MDA-MB-231 cells (Figure 1f). Correlating with this, the EMT marker Slug was overexpressed in MDA-MB-231/IR cells (Figure 1e). These results indicate that MDA-MB-231/IR cells exhibit an enriched CSC population compared to the non-irradiated parental MDA-MB-231 cells.

### 2.2. MDA-MB-231/IR Cells Exhibited Low ROS Levels

**Figure 2:**
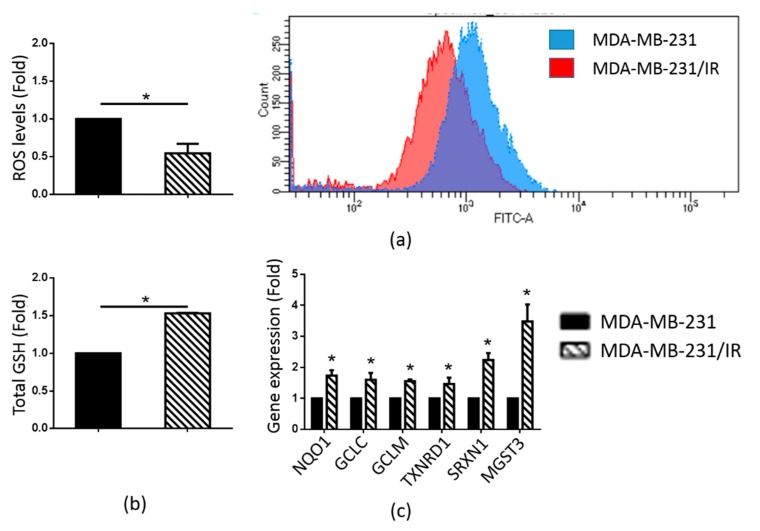
ROS and GSH levels and expression of genes related to antioxidant effects in MDA-MB-231 and MDA-MB-231/IR cells. (**a**) ROS levels were evaluated after staining with H_2_DCFDA. (**b**) Total glutathione levels were measured by the GSH assay. (**c**) The expression of antioxidant-related genes was analyzed by real-time PCR; * *p* < 0.05; results are presented as mean ± standard deviation.

Numerous studies have shown that CSCs possess increased ROS scavenging activities, leading to lower ROS levels than the levels in cancer cells [5]. We found that the ROS levels in MDA-MB-231/IR cells were 1.83-fold lower than in the parental cells (Figure 2a). A glutathione (GSH) assay was performed to measure the GSH levels in the two cell lines, since GSH has been reported to be a major ROS scavenger [41]. As shown in Figure 2b, the MDA-MB-231/IR cells exhibited higher GSH levels. Correlating with these results, there was increased expression of antioxidant genes [42] such as NAD(P)H quinone oxidoreductase 1 (NQO1), glutamate-cysteine ligase catalytic subunit (GCLC), glutamate-cysteine ligase modifier subunit (GCLM), thioredoxin reductase 1 (TXNRD1), sulfiredoxin 1 (SRXN1), and microsomal glutathione S-transferase 3 (MQST3) (Figure 2c). These results demonstrate that, in contrast to MDA-MB-231 cells, MDA-MB-231/IR cells maintained low ROS levels due to their higher expression of ROS scavengers.

### 2.3. MTDH Expression is Significantly Correlated with Poorer Prognosis in Breast Cancer Patient Samples

**Figure 3:**
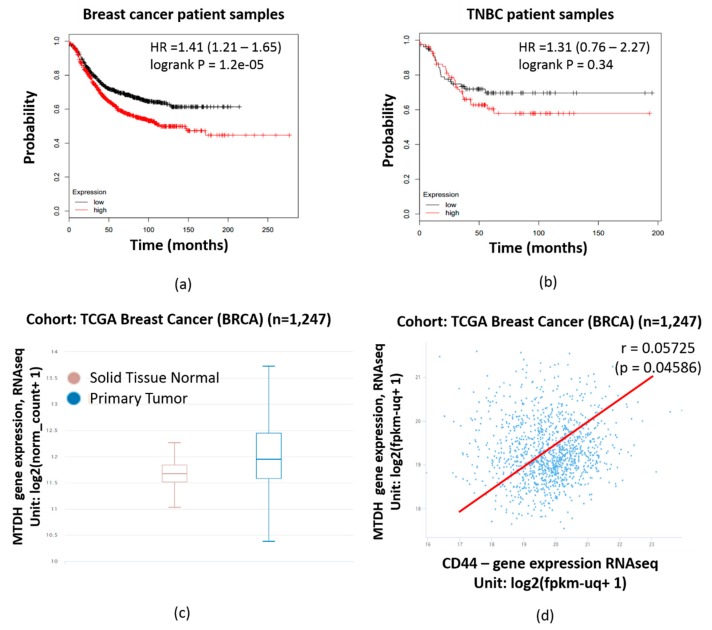
Analysis of metadherin (MTDH) expression in breast cancer patients. Correlation between MTDH expression and survival rate in total breast cancer patients (**a**) and triple negative breast cancer (TNBC) patients (**b**), as analyzed by Kaplan–Meier plotting. (**c**) MTDH expression in primary tumor and normal tissue in the The Cancer Genome Atlas (TCGA) breast cancer (BRCA) cohort. (**d**) Correlation between MTDH and CD44 expression, in the TCGA breast cancer (BRCA) cohort; * *p* < 0.05; results are presented as mean ± standard deviation.

Kaplan–Meier plot analysis was performed to examine the correlation between MTDH expression and the survival rates of breast cancer and TNBC patients. Higher expressions of MTDH and shorter lifetimes were found to be correlated in breast cancer and TNBC patients, as shown in Figure 3a,b, respectively. Moreover, Xena browser analysis indicated a higher expression of MTDH at the mRNA level in primary tumor samples compared to normal tissue (Figure 3c) (*n* = 1247, *p*-value = 7.699 × 10^−9^, f = 18.97). Xena browser analysis also revealed a positive correlation between the MTDH and CD44 expression levels in The Cancer Genome Atlas (TCGA) breast cancer database (Figure 3d) (*n* = 1247, r = 0.05725, *p*-value = 0.04586). The positive correlation of MTDH with poor prognosis in breast cancer patients suggests that MTDH is an ideal target for chemotherapy.

### 2.4. MTDH Plays a Key Role in Maintenance of the CSC Population in MDA-MB-231/IR Cells

**Figure 4:**
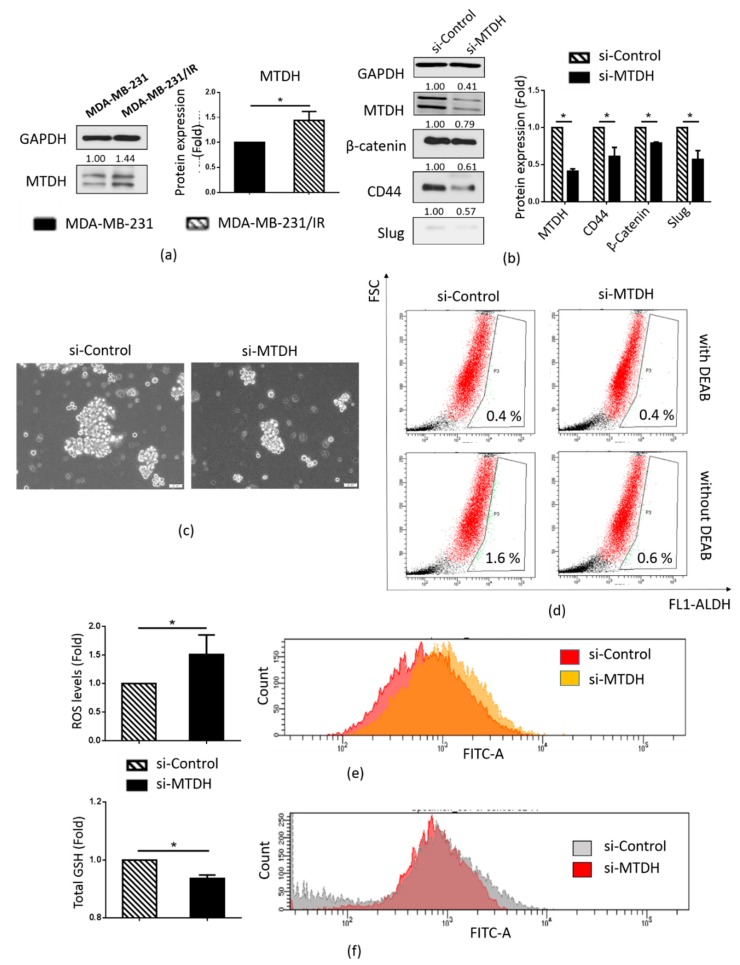
Role of MTDH in regulating the CSC population in MDA-MB-231/IR cells. (**a**) Western blot analysis of MTDH in MDA-MB-231 and MDA-MB-231/IR cells. (**b**) Western blot analysis of MTDH, β-catenin, CD44, and Slug after knockdown by si-MTDH for 48 h. (**c**) Appearance of mammospheres after siRNA knockdown (100× magnification). (**d**) ALDH^+^ population obtained by the ALDEFLUOR assay kit, with DEAB used as a negative control. (**e**) ROS levels measured by FACS analysis and H_2_DCFDA staining. (**f**) The CD44^+^ population, as assessed by FACS analysis; * *p* < 0.05; results are presented as mean ± standard deviation.

As MTDH has been reported to play a prominent role in breast cancer therapy resistance [33,39] and maintenance of the CSC population [31], we examined whether MTDH could play a role in the stemness of MDA-MB-231/IR cells. We found that MTDH was overexpressed (1.44-fold) in MDA-MB-231/IR cells compared to the parental MDA-MB-231 cells (Figure 4a). Figure 4b shows that the knockdown of MTDH resulted in a decrease in the MTDH protein levels (2.43-fold reduction) compared to the si-RNA control, and inhibition of MTDH expression in MDA-MB-231/IR cells resulted in reductions in mammosphere formation (Figure 4c) and ALDH activity (Figure 4c,d). These results were supported by decreases in CSC markers such as β-catenin and Slug (Figure 4b). Moreover, increased ROS production was observed (Figure 4e), as well as reductions in both total CD44 expression (Figure 4b) and CD44 expression on cell surfaces (Figure 4f) following the knockdown of MTDH. These results show that MTDH is responsible for the maintenance of the CSC population in MDA-MB-231/IR cells.

### 2.5. PEITC Reduced MDA-MB-231/IR Cell Viability by Inducing ROS Accumulation

**Figure 5:**
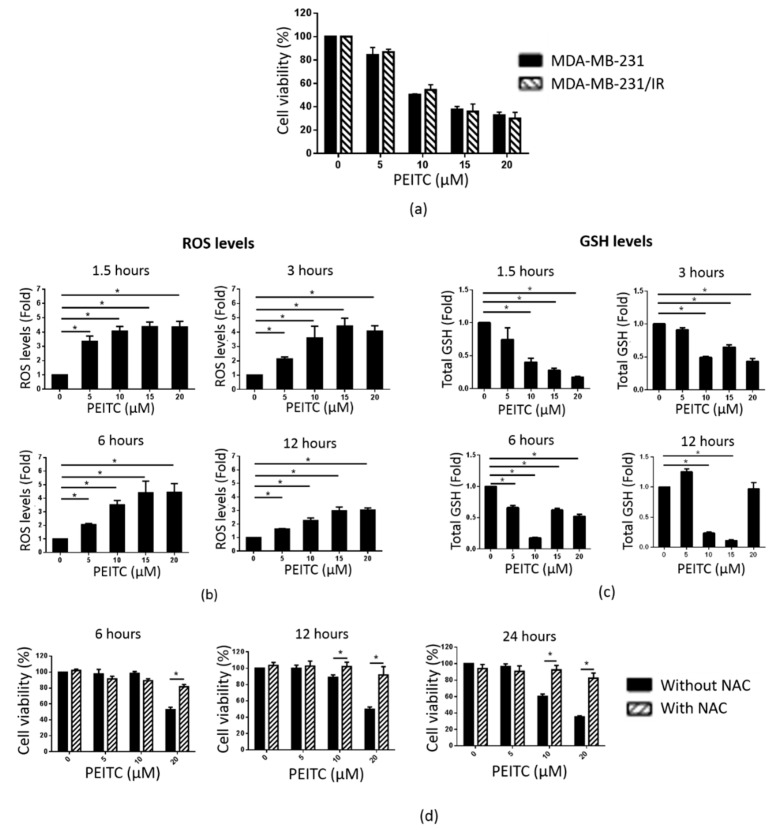
PEITC reduces cell viability in MDA-MB-231/IR cells by increasing ROS levels. (**a**) Cell viability was assessed by the MTT assay. (**b**) ROS levels were analyzed after staining with H_2_DCFDA. (**c**) Total GSH levels were measured by a GSH assay. (**d**) Pre-treatment with NAC (10 mM) helped maintain the viability of MDA-MB-231/IR cells, as assessed by the MTT assay; * *p* < 0.05; results are presented as mean ± standard deviation.

An MTT assay was performed to compare the effects of PEITC on the proliferation of MDA-MB-231 and MDA-MB-231/IR cells. As shown in Figure 5a, PEITC reduced the viability of MDA-MB-231/IR cells, with an IC_50_ of 9.29 μM. PEITC exerted non-cytotoxic effects in MCF-10A (mammary epithelial cells) and fibroblast cells (Figure S1). As PEITC has been reported to be a pro-oxidant substance due to its conjugation with GSH [41], we measured ROS and GSH levels in MDA-MB-231/IR cells after exposure to PEITC at different time points (1.5, 3, 6, and 12 h). As seen in Figure 5b, the ROS levels were increased in a dose-dependent manner at each time point tested, following a sharp enhancement in the expression of antioxidant genes (Figure S2). Correlating with these results, GSH levels were reduced at 1.5, 3, and 6 h post incubation (Figure 5c). N-Acetylcysteine (NAC), a ROS scavenger [43], was used to examine whether the cytotoxic effects of PEITC were caused by elevated ROS production. Co-treatment with NAC and PEITC allowed for the proliferation of MDA-MB-231/IR cells, confirming that the PEITC-induced reduction of MDA-MB-231/IR cell proliferation was due to the increased ROS levels (Figure 5d). These data demonstrate that PEITC can selectively target MDA-MB-231/IR cells by promoting ROS accumulation.

### 2.6. PEITC Reduced Stemness and the Expression of MTDH and CSC Markers in MDA-MB-231/IR Cells

**Figure 6:**
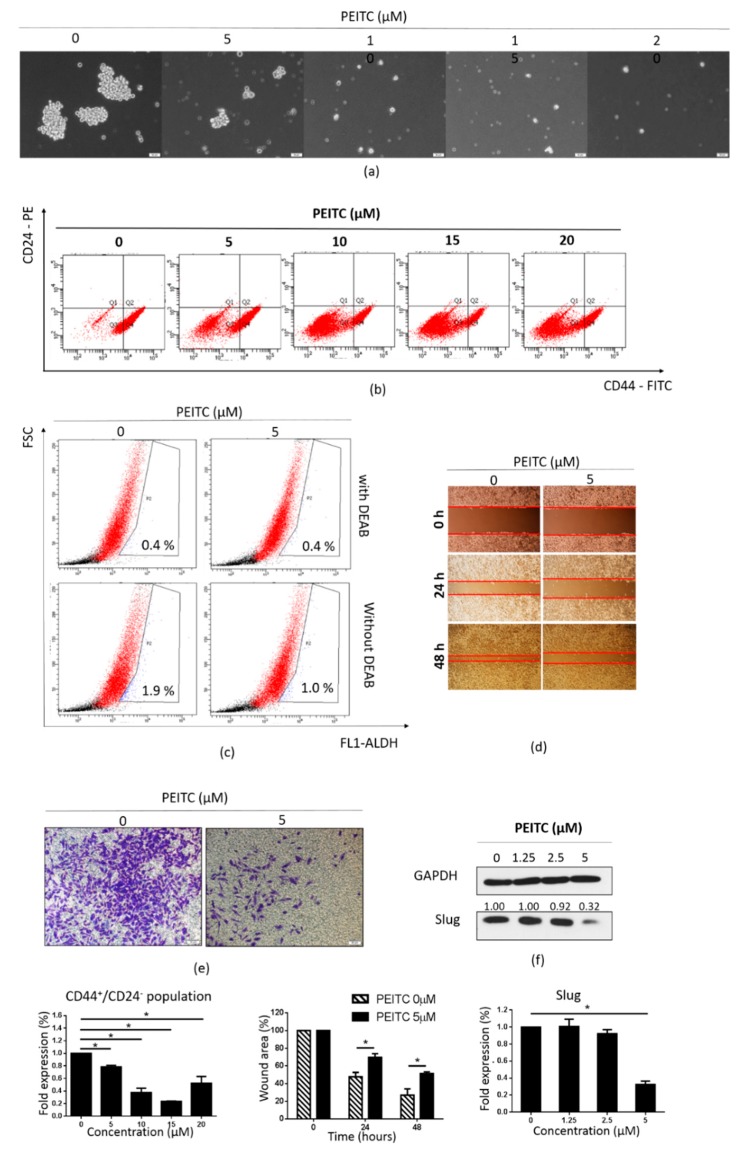
Effects of PEITC on stemness in MDA-MB-231/IR cells. (**a**) Mammospheres were cultured in complete MammoCult Human Medium (100× magnification). (**b**) CD44^+^/CD24^−^ populations were assessed by FACS analysis after incubation with PEITC (0, 5, 10, 15, or 20 μM) for 24 h. (**c**) The ALDH^+^ population was examined using the ALDEFLUOR assay kit, with DEAB used as a negative control; cells were treated with PEITC (0 or 5 μM) for 24 h. (**d**) Cell migration was determined by the wound healing assay; cells were treated with PEITC (0 or 5 µM) for 24 or 48 h (100× magnification). (**e**) Invasive cells were stained with crystal violet after treatment with PEITC (0 or 5 µM) for 24 h (100× magnification). (**f**) Western blot analysis of Slug after incubation of cells with PEITC (0, 1.25, 2.5, or 5 μM) for 24 h; * *p* < 0.05; results are presented as mean ± standard deviation.

**Figure 7:**
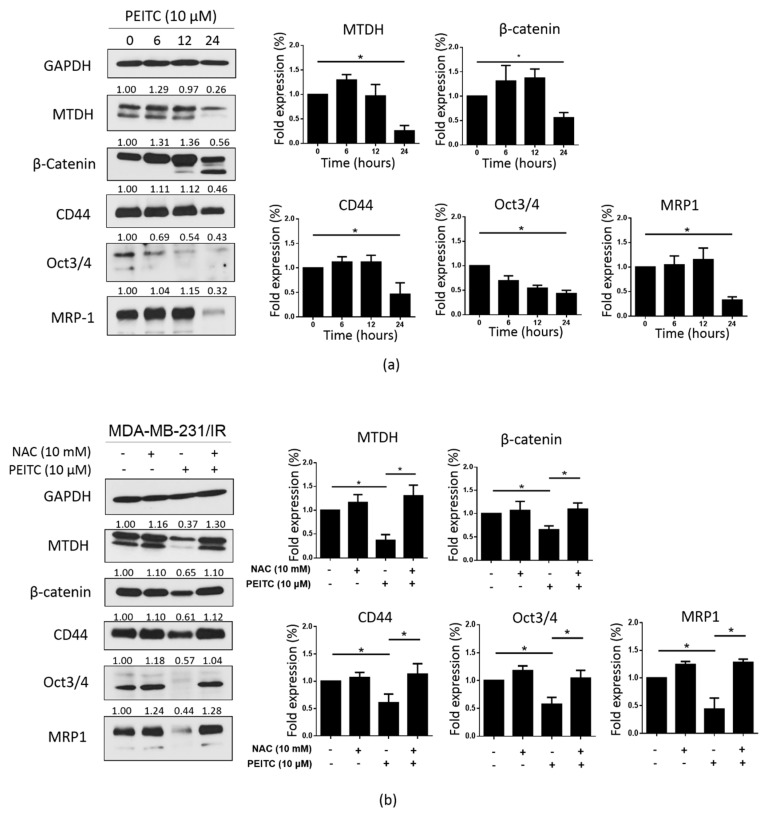
PEITC targets MTDH and CSC markers in MDA-MB-231/IR cells. (**a**) The levels of MTDH and CSC markers were assessed by Western blotting following PEITC (0, 10 μM) treatment. (**b**) NAC pre-treatment helped maintain the expression of MTDH and CSC markers; * *p* < 0.05; results are presented as mean ± standard deviation.

Next, we evaluated whether PEITC could suppress the CSC population of MDA-MB-231/IR cells. PEITC dramatically reduced the formation of mammospheres in MDA-MB-231/IR cells after exposure for 24 h at the doses shown in Figure 6a. In addition, the CD44^+^/CD24^−^ population was decreased in a dose-dependent manner after PEITC treatment (Figure 6b). Inhibition of ALDH activity and the suppression of cell migration and invasion at non-lethal concentrations of PEITC were also apparent (Figure 6c–e). These observations were further supported by Western blot analysis, in which reduced expression of Slug was observed (Figure 6f). Next, we examined the effect of PEITC on the expression of MTDH and CSC markers. As seen in Figure 7a, PEITC significantly reduced the expression of MTDH and CSC markers in a time-dependent manner. Co-treatment with NAC prevented the decrease in the expression of these proteins triggered by PEITC (Figure 7b). All of these data demonstrate that PEITC effectively reduced CSCs and CSC markers in MDA-MB-231/IR cells, accompanied by decreased MTDH levels.

### 2.7. PEITC Downregulated MTDH at the Post-Transcriptional Level

**Figure 8:**
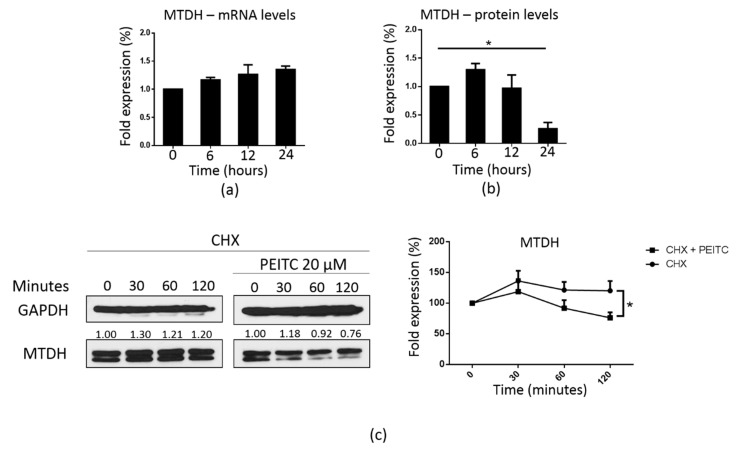
PEITC regulates MTDH post-transcriptionally. (**a**) Real-time PCR analysis of MTDH following PEITC treatment (10 µM for 6, 12, or 24 h). (**b**) Western blot analysis was performed to analyze MTDH expression following PEITC exposure (10 µM for 6, 12, or 24 h), similar to Figure 7a. (**c**) Western blot analysis of the MTDH protein expression following co-treatment with PEITC (20 µM) and cycloheximide (100 μg/mL) for 30, 60, or 120 min; * *p* < 0.05; results are presented as mean ± standard deviation.

We further investigated the underlying mechanism associated with the suppression of MTDH induced by PEITC. Interestingly, there was no reduction in the *MTDH* mRNA levels, but the levels of the MTDH protein were dramatically reduced after treatment with 10 μM PEITC for 24 h (Figure 8a,b). Based on these results, we hypothesized that PEITC could regulate MTDH at the post-transcriptional level. To confirm this, the MTDH levels were measured by Western blotting after cells were treated with cycloheximide, a ribosome inhibitor [44], with or without PEITC. As shown in Figure 8c, compared to cells treated with cycloheximide alone, cells treated with both cycloheximide and PEITC exhibited a dramatic reduction in the expression of MTDH after incubation for 120 min, indicating that the expression of MTDH is regulated at the post-transcriptional level.

## 3. Discussion

CSCs play a major role in chemo and radio-resistance in breast cancer patients [45]. In a recent study, we generated a chemo and radio-resistant MDA-MB-231/IR cell line that exhibited enhanced CSC characteristics in comparison to its parental cell line MDA-MB-231 [40]. However, the underlying mechanisms responsible for this phenomenon are still unclear. In this study, we showed that MDA-MB-231/IR cells have typical stem cell characteristics such as increased mammosphere formation capacity, increased CD44^+^/CD24^−^ populations, enhanced expression of CSC markers such as CD44, Oct3/4, Slug, and MRP1, and enhanced invasion capability (Figure 1b–f). In addition, we examined ALDH activity since ALDH has widely been used as a CSC marker in BCSCs [4]. We found that MDA-MB-231/IR cells exhibited higher ALDH activity than MDA-MB-231 cells, thereby reaffirming the stemness of the MDA-MB-231/IR cells. Furthermore, as lower ROS levels have been recognized as a hallmark feature of CSCs, and ROS are crucial mediators of radiation-induced cell death [5], we tested whether MDA-MB-231/IR cells accumulated lower ROS levels than MDA-MB-231 cells. Our results showed that the ROS levels were reduced in MDA-MB-231/IR cells, and this was associated with higher expressions of ROS scavengers such as GSH [43] (Figure 2b) and antioxidant genes (Figure 2c). These results suggest that ROS are ideal targets for CSC-targeted therapies [6,7,8].

We further identified the underlying mechanisms associated with the enrichment of the CSC population in MDA-MB-231/IR cells. It has been reported that MTDH plays a key role in cancer therapy resistance due to its ability to maintain CSC populations [14,31,35]. Elevated expressions of MTDH are associated with poor prognoses in breast cancer and TNBC patients (Figure 3a,b). Additionally, MTDH has been found to be more highly expressed in primary tumor tissues than in normal tissues (Figure 3c). Moreover, previous studies have shown that MTDH can regulate the NF-κB pathway [34,35] and MTDH is induced by the TNF-α pathway [46]. Our previous study revealed that MDA-MB-231/IR cells show enhanced activity in the NF-κB pathway, the TNF signaling pathway, and the Toll-like receptor pathway [40]. Xena browser analysis revealed a positive correlation between MTDH and the BCSC marker CD44 in breast cancer patients (Figure 3d). In light of these findings, we hypothesized that MTDH plays a critical role in the stemness of MDA-MB-231/IR cells. Western blot analysis revealed an increase in MTDH expression in MDA-MB-231/IR cells (Figure 4a). Then, to confirm the role of MTDH in maintaining the CSC population in this line, MTDH was knocked down using si-MTDH. Successful knockdown of MTDH (Figure 4b) resulted in a reduction in the CSC population, as evidenced by reduced mammosphere formation capacity (Figure 4c) and ALDH activity (Figure 4d). Western blot results (Figure 4b) showing decreased levels of CSC markers, such as β-catenin, CD44, and Slug, strongly support our data. The CD44-positive population was also reduced after knockdown of MTDH (Figure 4f). Numerous studies have reported that MTDH can promote CSC characteristics in colorectal cancer [47], glioblastoma cancer [31], pancreatic cancer [48], and breast cancer [49,50,51]. Moreover, Hu et al. (2017) revealed that MTDH contributes to the maintenance of glioblastoma CSCs by protecting β-catenin from phosphorylation and degradation by proteasomes. In addition, β-catenin translocates into the nucleus and acts as a transcription factor to activate CSC-related downstream genes [31]. Knockdown of MTDH also resulted in enhanced ROS levels (Figure 4e). It has been reported that MTDH knockdown can induce ROS production, and MTDH overexpression has resulted in reduced ROS levels [38].

It is well-established that PEITC promotes ROS accumulation by covalently binding to GSH [41,52]. Since MDA-MB-231/IR cells exhibited increased ROS scavenger machinery, including GSH and antioxidant genes, resulting in very low ROS levels (Figure 2a), we hypothesized that by inducing ROS accumulation, PEITC could target the CSC population in MDA-MB-231/IR cells. Our results showed that PEITC could eliminate the CSC population in MDA-MB-231/IR cells, as evidenced by the significant inhibition of mammosphere formation, the CD44^+^/CD24^−^ population, and ALDH activity (Figure 6a–c). PEITC has been reported to target CSC compartments in a variety of cancers, including cervical cancer [53], colon cancer [33], colorectal cancer [54], and HeLa cancer cells [10]. Recently, Koschorke et al. (2019) found that PEITC can target HER-2 positive BCSCs, and this effect was enhanced in combination with trastuzumab therapy [55]. Moreover, Western blot analysis revealed that PEITC can reduce levels of CD44, Oct3/4, and MRP-1, which are widely used as BCSC markers (Figure 7a). In addition, the levels of β-catenin, a transcription factor that contributes to the expression of many CSC genes [31], were significantly reduced after incubation with PEITC (Figure 7a). Chen et al. (2018) reported that PEITC significantly inhibits sphere formation in colorectal cancer by targeting the Wnt/β-catenin pathway [54]. Importantly, incubation with PEITC promoted ROS production (Figure 5b) and decreased GSH levels (Figure 5c). However, during longer incubations with PEITC, decreased ROS levels were detected (Figure 5b). This can be explained by the complicated activities of ROS scavengers inside the cells. In addition to GSH, antioxidant genes can also control ROS levels. The rapidly elevated ROS levels after PEITC treatments of just 1.5, 3, and 6 h activated the expression of antioxidant genes (Figure S2). We then used NAC, a known ROS scavenger [43], to confirm our hypothesis. Co-treatment with PEITC and NAC prevented the changes in cell death (Figure 5d) and CSC markers (Figure 7b) that are induced by PEITC. Indeed, through enhanced ROS accumulation, PEITC could erase the CSC characteristics induced by irradiation in MDA-MB-231 cells.

Ours is the first report to show that PEITC can target MTDH at the post-transcriptional level in radiation-induced CSCs of TNBC cells. We found that, while there was no significant change in MTDH expression at the mRNA level, the levels of MTDH protein were dramatically reduced after 24 h of exposure to 10 μM PEITC (Figure 8a,b). Moreover, co-treatment with cycloheximide, a ribosome inhibitor [44], dramatically reduced MTDH expression in MDA-MB-231/IR cells after incubation for 120 min (Figure 8c). These data strongly suggest that PEITC regulates MTDH at the post-transcriptional level. One of the best-known mechanisms supporting this phenomenon is the ubiquitin-proteasome proteolytic pathway. Once a protein is conjugated with a poly-ubiquitin chain, it is recognized and degraded by the proteasome system [56]. PEITC has been reported to induce ubiquitination through the inhibition of deubiquitinase activity [57]. MicroRNAs may also play a role in the regulation of MTDH expression by PEITC. MicroRNAs are groups of small RNAs (−22 nucleotides in length) that function by binding to complementary target sequences of messenger RNAs. [58]. The TargetScan Human analysis software (Figure S3) revealed that MTDH is regulated by several microRNAs [59]. The microRNAs miR-26, miR-142, and miRNA-135 have been identified as possible targets of PEITC [60]. These findings provide a strong rationale to further investigate the mechanistic roles of the ubiquitin-proteasome proteolytic pathway as well as the PEITC-targeted miRNAs in the post-transcriptional regulation of MTDH by PEITC. In-depth follow-up studies are needed to determine the specific mechanisms that regulate the expression of MTDH. In summary, our study demonstrates, for the first time, that PEITC can target MTDH in MTDH-overexpressing radioresistant MDA-MB-231/IR cells by increasing ROS production.

It is has reported that PEITC can exert anti-metastatic effects in a breast cancer cell model [61]. Moreover, PEITC has enhanced the efficacy of chemotherapy drugs such as doxorubicin and paclitacxel in breast cancer cells [61]. Moreover, PEITC treatment has been reported to inhibit breast tumor growth via suppressing T regulatory lymphocytes [61]. In a recent publication, PEITC has been shown to inhibit the activity of proteasomal cysteine deubiquitinases UCHL5 [62]. Through depleting GSH levels, PEITC sensitized breast cancer cells to radiation by modulating cellular metabolism [63]. Growing evidence of in-vitro therapeutic efficacy of cruciferous vegetables derived isothiocyanates or extracts has provided a strong rational to investigate their therapeutic potential in several clinical studies. Among isothiocyanates, phenethyl isothiocyanate and sulforaphane have provided promising results in some cancer and autism clinical trials. Apart from cancer and autism, cruciferous vegetables derived isothiocyanates or extracts have been clinically investigated for skin disorders, diabetes, heart diseases, and respiratory conditions [64]. Clinical trials of cruciferous vegetables derived isothiocyanates are extremely limited in breast cancer. A study conducted by Atwell el al. in 2015 showed that glucoraphanin, a well-known broccoli glucosinolate, can mediate epigenetics alterations in peripheral blood mononuclear cells without producing significant alterations in breast cancer tissue tumor biomarkers [65]. New findings of the present investigation in radiotherapy resistant triple negative breast cancer cells will provide a rational basis for conducting future clinical investigations with phenethyl isothiocyanate in breast cancer patients.

## 4. Materials and Methods

### 4.1. Cell Culture

Human TNBC cells (MDA-MB-231) and normal mammary epithelial cells (MCF-10A) were purchased from the American Type Culture Collection (ATCC, Rockville, MD, USA). The radioresistant MDA-MB-231/IR cell line was established and characterized as previously described [40]. Normal fibroblast cells were a gift from Prof. Moonjae Cho. MDA-MB-231 and MDA-MB-231/IR cells were cultured in Dulbecco’s modified Eagle’s medium (DMEM) with 10% heat-activated fetal bovine serum (FBS), 100 U/mL penicillin, and 100 µg/mL streptomycin. MCF-10A cells were cultured under the ATCC-recommended culture conditions. All cells were maintained at 37 °C with a 5% CO_2_ atmosphere.

### 4.2. Cell Viability Assay

Cells (2 × 10^4^/mL) were seeded in 96-well plates for 24 h and treated with PEITC (Sigma, St. Louis, MO, USA). Following 24 h of incubation, 100 µL of MTT (0.5 mg/mL) dissolved in fresh medium was added to each well, and the plates were re-incubated for 2–3 h. To dissolve the formazan crystals, the medium was aspirated and 150 µL of dimethyl sulfoxide (DMSO) was added to each well. A microplate reader (Tecan Group, Ltd., Salzburg, Austria) was used to record the absorbance at 570 nm. The percentage of cell viability was calculated using the formula (control group − treated group) ÷ control group) × 100%.

### 4.3. Cell Migration Assay

For the cell migration assay, 6-well cell culture plates were used. Initially, 1 × 10^5^ cells were seeded and incubated for 24 h. Upon 90% confluency, scratches were manually made using a sterile pipette tip. The cells were then washed with phosphate-buffered saline (PBS) to remove detached cells and incubated with or without PEITC (0, 5 μM). Wound areas were photographed with the help of an inverted phase-contrast microscope at 4× magnification.

### 4.4. Cell Invasion Assay

A Transwell system (24-well plate; Corning, Cambridge, MA, USA) was used to examine cell invasion. In the upper chamber, matrigel was coated and cells (1.5 × 10^5^ per Transwell) were seeded in 200 µL of serum-free medium, supplemented with or free of, PEITC. The lower chamber was filled with 750 µL of DMEM supplemented with 10% FBS. Following 24 h of incubation, invading cells were fixed with 4% paraformaldehyde followed by methanol. Finally, the cells were stained with 2% crystal violet and were observed under a phase-contrast microscope.

### 4.5. Flow Cytometric Analysis of the CD44^+^/CD24^−^ Population

Fluorescence-activated cell sorting (FACS) was conducted to analyze the expression of the CD44 and CD24 markers. Briefly, 1 × 10^6^ cells were suspended in 100 μL of immunofluorescence staining buffer supplemented with PE-conjugated anti-human CD24 antibody (BD Pharmingen, San Diego, CA, USA) and FITC-conjugated anti-human CD44 antibody (BD Pharmingen) and incubated for 10 min at 4 °C. Following incubation, the cells were washed with PBS and the CD44^+^/CD24^−^ cell population was determined using a FACSCalibur flow cytometer (Becton Dickinson, Franklin Lakes, NJ, USA).

### 4.6. Aldefluor Assay

The ALDEFLUOR assay kit (Stemcell Technologies, Vancouver, BC, Canada) was used to measure ALDH enzyme activity. Diethylaminobenzaldehyde (DEAB), a specific inhibitor of ALDH, was used as the negative control. Prior to the assay, 3 × 10^4^ cells/mL were cultured in 60 mm dishes and incubated for 24 h. Following incubation, the cells were exposed to PEITC (5 μM, non-toxic concentration) for 24 h, and then the ALDEFLUOR assay was performed. The samples were analyzed for ALDH-positive cells using a FACSCalibur flow cytometer.

### 4.7. Mammosphere Formation Assay

For the mammosphere formation assay, 2 × 10^4^ cells/mL were seeded as single cells in ultralow-attachment dishes containing complete MammoCult Human Medium (Stemcell Technologies) supplemented with or without PEITC (0, 5, 10, 15, or 20 μM). After 7 days of incubation, mammospheres were observed (larger than 60 μm) under a phase-contrast microscope.

### 4.8. Real-Time PCR

Total RNA was extracted using TRIzol reagent (Invitrogen, Carlsbad, CA, USA). Following reverse transcription, two-step quantitative real-time PCR was performed (Takara, Shiga, Japan). The primers used for real-time PCR experiments are listed in Table S1. GAPDH, a housekeeping gene was used as an internal control. The results are expressed using the formula 2−ΔΔCq [66].

### 4.9. Western Blot Analysis

Western blot analysis was conducted, as previously described [67]. Briefly, cell lysates were prepared using radioimmunoprecipitation assay (RIPA) buffer and quantified using a commercially available kit. After determining the protein concentrations, 20–40 µg of proteins were separated using sodium dodecyl sulfate polyacrylamide gel electrophoresis and were electrophoretically transferred to PVDF membrane. After blocking with skim milk, the membranes were exposed to different primary antibodies. Except for the anti-GAPDH primary antibody (1:7000 dilution), the primary antibodies were diluted 1:1000. All primary antibodies were purchased from the Cell Signaling Technology (Beverly, MA, USA). The anti-rabbit immunoglobulin G (IgG) secondary antibody (Vector Laboratories, Burlingame, CA, USA) was diluted 1:5000 prior to use. Bands were detected using the BS ECL Plus Kit (Biosesang, Seongnam, South Korea). ImageJ software (US National Institutes of Health, Bethesda, MD, USA) was used to quantify bands.

### 4.10. siRNA Knockdown

To knock down MTDH, a small interfering RNA specific for MTDH (si-MTDH) and a scrambled siRNA control (si-Control) were purchased from Santa Cruz Biotechnology (Dallas, TX, USA). Cells were seeded at 5 × 10^4^ cells/well in 6-well plates and incubated for 24 h. Then, the cells were transfected for 48 h with si-MTDH using the Lipofectamine 3000 reagent (Invitrogen) according to the manufacturer’s instructions. The protein expression levels after MTDH knockdown were determined by Western blotting.

### 4.11. ROS Generation Analysis

Prior to the assay, 3 × 10^6^ cells/mL were seeded in 60 mm culture dishes. After 24 h of incubation, the cells were treated with or without PEITC and re-incubated for 1.5, 3, 6, or 12 h. To analyze ROS generation, the cells were stained with 2′,7′-dichlorofluorescin diacetate (H_2_DCFDA) for 15 min, followed by washing with 2 mL of PBS. A FACSCalibur flow cytometer was used to assess the ROS levels.

### 4.12. GSH Assay

GSH activity was measured using a GSH assay kit (Cayman Chemical, Ann Arbor, MI, USA). Cells (3 × 10^6^ cells/mL) were cultured in 60 mm culture dishes and incubated for 24 h. After incubation, the cells were exposed to different concentrations of PEITC (0, 5, 10, 15, or 20 μM) for 1.5, 3, 6, or 12 h. GSH activity was measured according to the manufacturer’s instructions.

### 4.13. Correlation Analysis by the Kaplan–Meier Plotter

The Kaplan–Meier Plotter database was used to examine the correlation between the expression of MTDH and the survival of rate of breast cancer patients. The UCSC Xena browser was used to examine possible associations in the expression of MTDH and CD44 in breast cancer patient samples based on the data available in the TCGA database.

### 4.14. Statistical Analysis

Data are presented as the mean ± standard deviation of at least three individual experiments. The data were statistically analyzed using the Student’s *t*-test with GraphPad software (La Jolla, CA, USA). A *p*-value < 0.05 was considered to indicate statistical significance.

## 5. Conclusions

In conclusion, we show here, for the first time, that MTDH plays a significant role in the regulation of stemness characteristics in radiation-induced MDA-MB-231/IR cells. PEITC is a well-known phytochemical that has been reported to elevate ROS in pre-clinical studies. In the present study, we demonstrated that PEITC can reduce the CSC population in MDA-MB-231/IR cells by upregulating the ROS levels. Furthermore, we determined that PEITC targets MTDH at the post-transcriptional level.

## Supplementary Materials

The following are available online at https://www.mdpi.com/2072-6694/12/2/268/s1. Table S1. Primer sequences of MTDH and anti-oxidant genes. Figure S1: MTT assay in the normal cell line MCF-10A and fibroblasts after PEITC treatment for 24 h. Figure S2: Real-time PCR analysis of antioxidant genes in MDA-MB-231/IR cells following PEITC treatment (10 µM for 1.5, 3, 6, 12, 24, or 48 h). Figure S3: Target genes of MTDH assessed by TargetScan Human analysis.
