# Supplementary material for: Phenethyl Isothiocyanate Suppresses Stemness in the Chemo- and Radio-Resistant Triple-Negative Breast Cancer Cell Line MDA-MB-231/IR Via Downregulation of Metadherin
Source: Cancers (Basel). 2020 Jan 22;12(2):268. doi: 10.3390/cancers12020268 (PMC7072670; doi:10.3390/cancers12020268)
Supplement: Supplementary file 1 [file cancers-12-00268-s001.pdf]

cancers

Supplementary Materials: Phenethyl Isothiocyanate Suppresses Stemness in the Chemo- and Radio-Resistant Triple-Negative Breast Cancer Cell Line MDA-MB-231/IR Via Downregulation of Metadherin

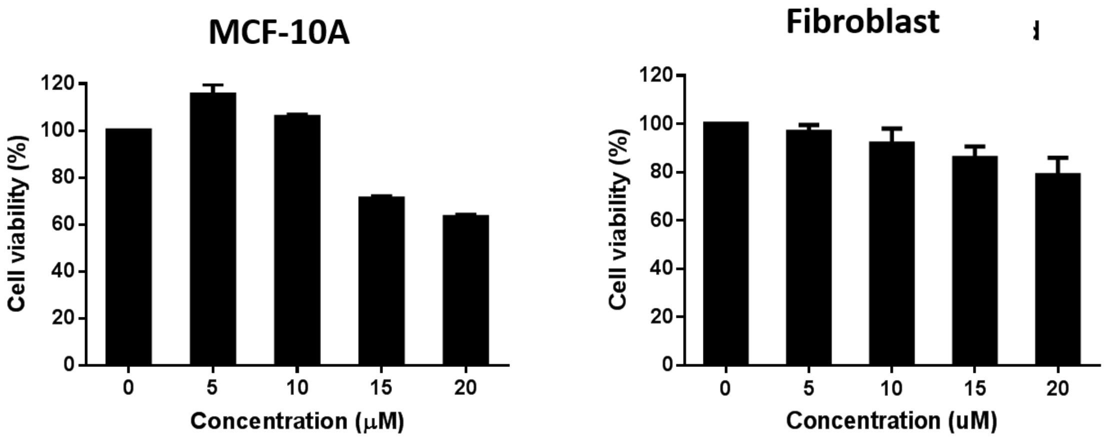

Figure S1. MTT assay in normal cell lines MCF-10A and Fibroblast after PEITC treatment for 24 h.

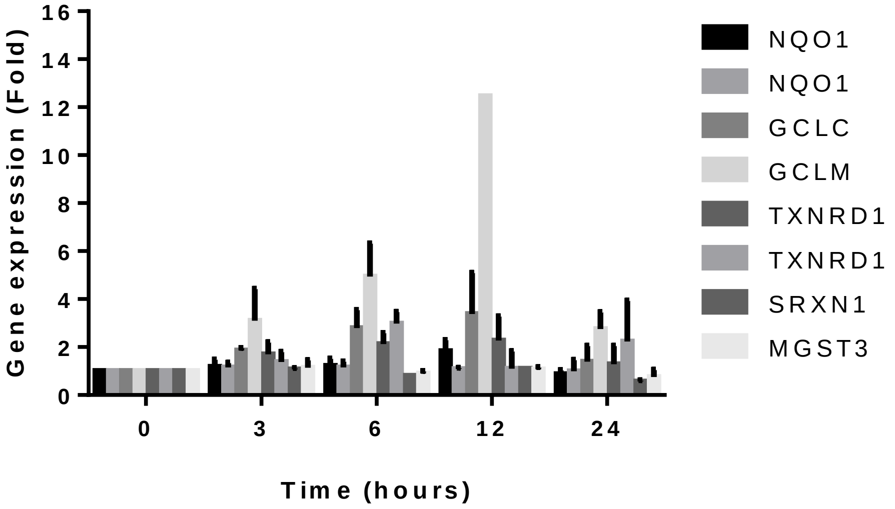

Figure S2. Realtime PCR analysis of anti-oxidant genes in MDA-MB-231/IR cells following PEITC treatment (10 μM for 3, 6, 12, and 24 h).

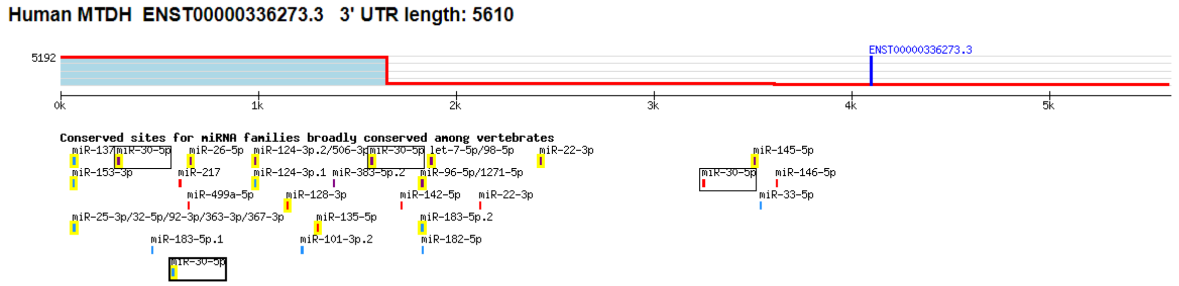

Figure S3. Target genes of MTDH assessed by TargetScan Human analysis.

Table S1. Primer sequences of MTDH and anti-oxidant genes.

Primer	Sequence
MTDH	5′- AAATAGCCAGCCTATCAAGACTC -3′<br>5′- TTCAGACTTGGTCTGTGAAGGAG -3′
NQO1	5′- GCAGACCTTGTGATATTCCAGT -3′<br>5′- GTCATACATGGCAGCGTAAGTG -3′
GCLC	5′- CTGGATGATGCTAATGAGTCTG -3′<br>5′- CTACTCTCCATCCAATGTCTGA -3′
GCLM	5′- CTAGACAAAACACAGTTGGAAC -3′<br>5′- ATGCAGTCAAATCTGGTGGCAT -3′
TXNRD1	Human Oxidative Stress Primer Library (RealTimePrimers.com)
SRXN1	Human Oxidative Stress Primer Library (RealTimePrimers.com)
MGST3	Human Oxidative Stress Primer Library (RealTimePrimers.com)

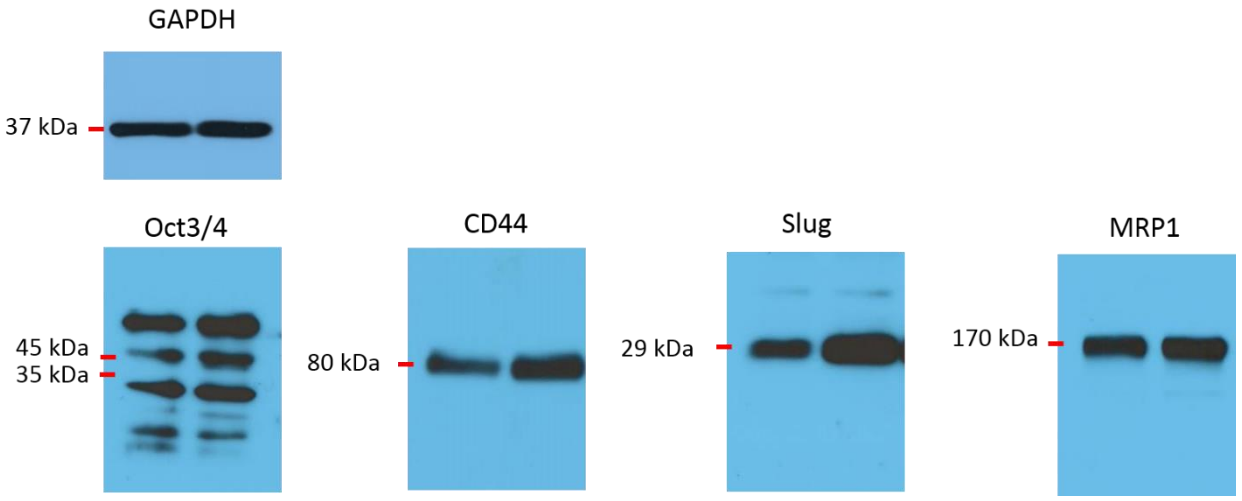

Figure S4. Original films of Figure 1e.

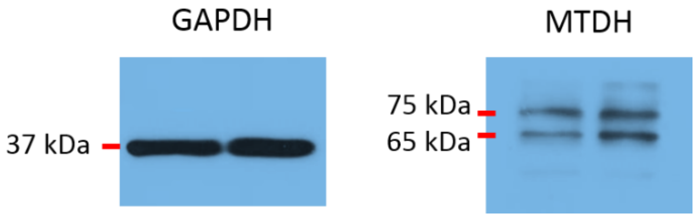

Figure S5. Original films of Figure 4a.

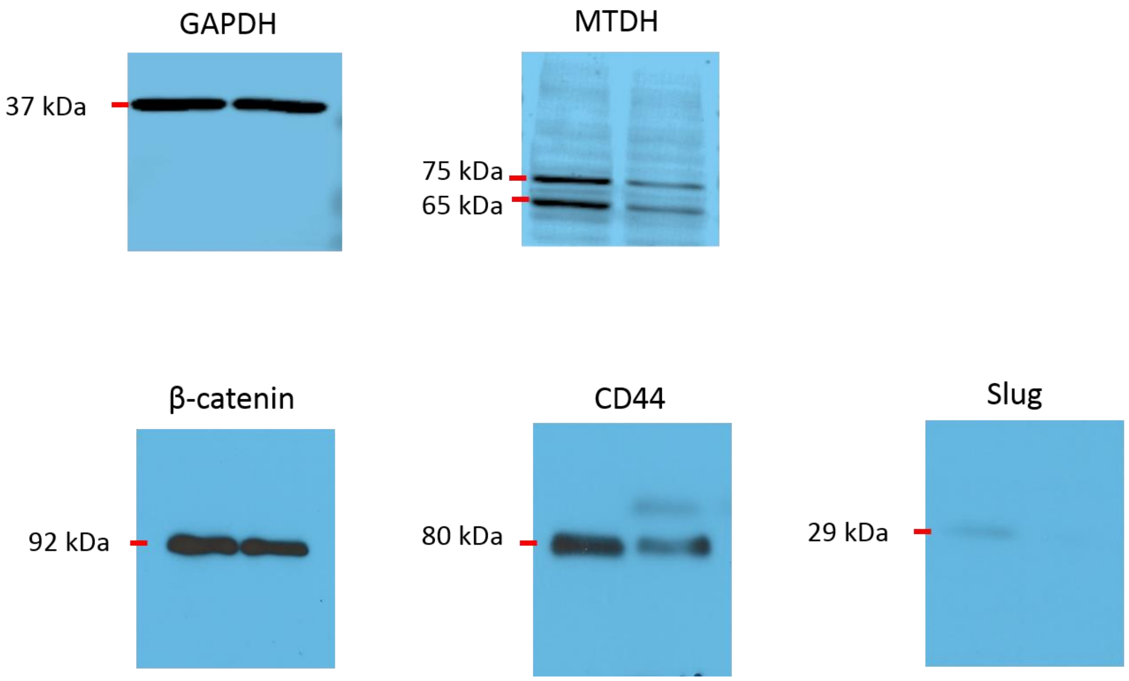

Figure S6. Original films of Figure 4b.

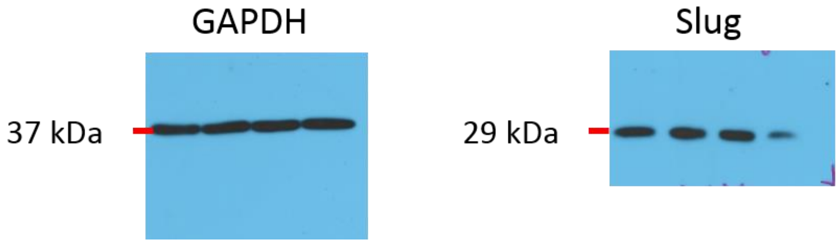

Figure S7. Original films of Figure 6f.

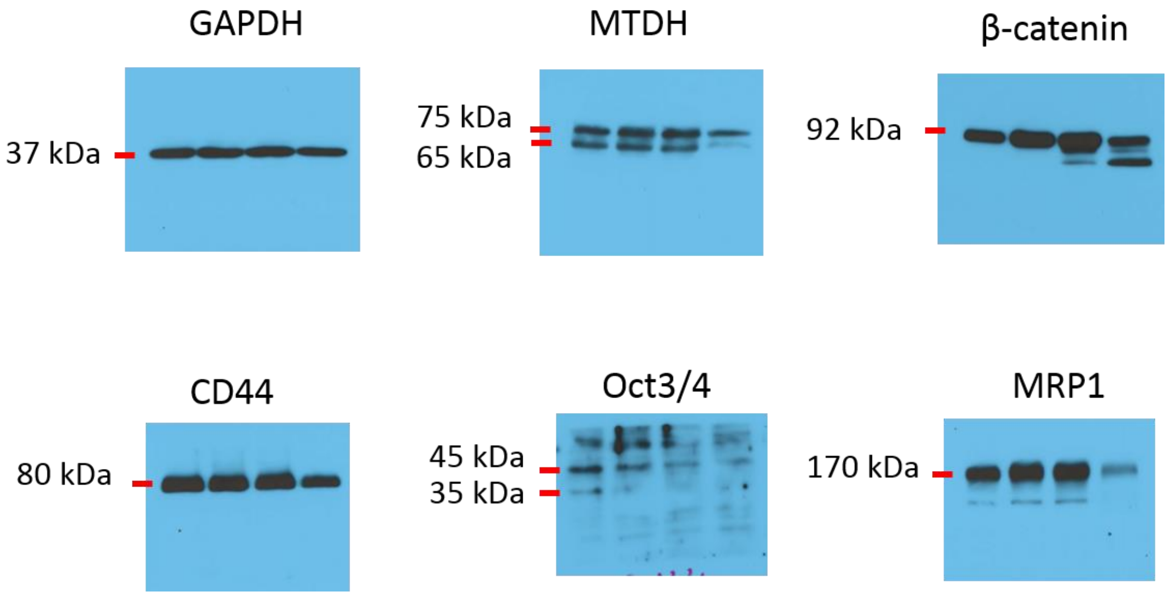

Figure S8. Original films of Figure 7a.

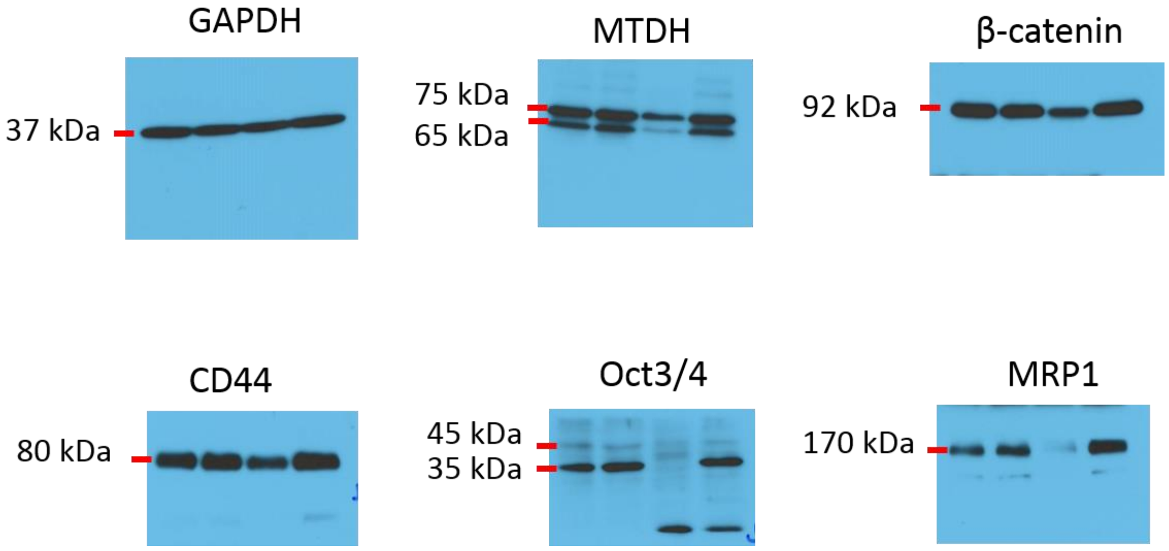

Figure S9. Original films of Figure 7b.

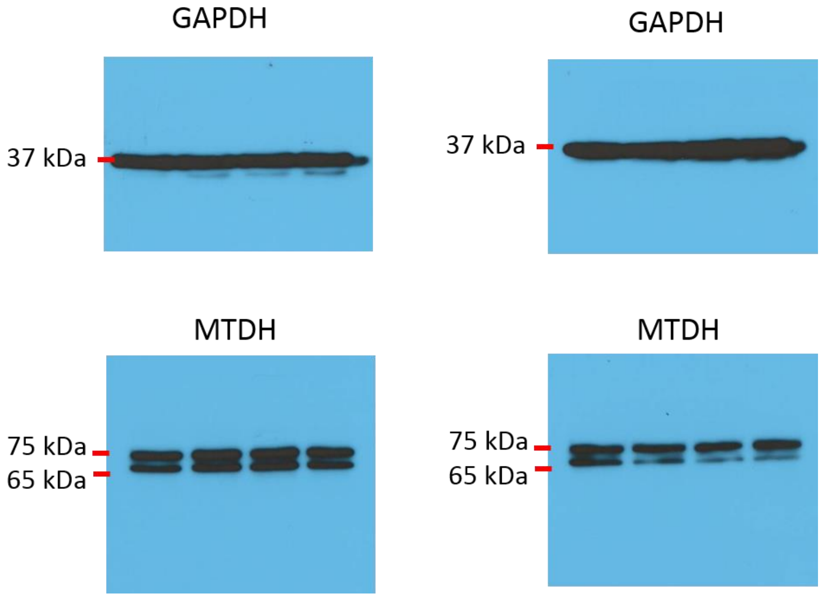

Figure S10. Original films of Figure 8c.

© 2019 by the authors. Submitted for possible open access publication under the terms and conditions of the Creative Commons Attribution (CC BY) license (http://creativecommons.org/licenses/by/4.0/).
